# Fat and Lean Mass Predict Bone Mass During Energy Restriction in Sedentary and Exercising Rodents

**DOI:** 10.3389/fphys.2018.01346

**Published:** 2018-09-25

**Authors:** Corinne E. Metzger, Sibyl N. Swift, Kyunghwa Baek, Mary Jane De Souza, Susan A. Bloomfield

**Affiliations:** ^1^Department of Health and Kinesiology, Texas A&M University, College Station, TX, United States; ^2^Department of Kinesiology, Pennsylvania State University, State College, PA, United States; ^3^Department of Physiology, Pennsylvania State University, State College, PA, United States

**Keywords:** energy restriction, treadmill running, bone mineral content, lean mass, fat mass

## Abstract

Energy restriction (ER) causes bone loss, but the impact of exercise during ER is less understood. In this study, we examined the impact of metabolic hormones and body composition on both total body bone mineral content (BMC) and local (proximal tibia) volumetric bone mineral density (vBMD) during short- (4 weeks) and long-term (12 weeks) ER with and without exercise in adult female rats. Our first goal was to balance energy between sedentary and exercising groups to determine the impact of exercise during ER. Second, we aimed to determine the strongest predictors of bone outcomes during ER with energy-matched exercising groups.

**Methods:** Female Sprague–Dawley rats were divided into three sedentary groups (*ad libitum*, –20% ER, and –40% ER) and three exercising groups (*ad libitum*, –10% ER, and –30% ER). Approximately a 10% increase in energy expenditure was achieved via moderate treadmill running (∼60–100 min 4 days/week) in EX groups. *n* per group = 25–35. Data were analyzed as a 2 × 3 ANOVA with multiple linear regression to predict bone mass outcomes.

**Results:** At 4 weeks, fat and lean mass and serum insulin-like growth factor-I (IGF-I) predicted total body BMC (*R*^2^ = 0.538). Fat mass decreased with ER at all levels, while lean mass was not altered. Serum IGF-I declined in the most severe ER groups (–40 and –30%). At 12 weeks, only fat and lean mass predicted total body BMC (*R*^2^ = 0.718). Fat mass declined with ER level regardless of exercise status and lean mass increased due to exercise (+5.6–6.7% vs. energy-matched sedentary groups). At the same time point, BMC declined with ER, but increased with exercise (+7.0–12.5% vs. energy-matched sedentary groups). None of our models predicted vBMD at the proximal tibia at either time point.

**Conclusion:** Both fat and lean mass statistically predicted total body BMC during both short- and long-term ER. Fat and lean mass decreased with ER, while lean mass increased with EX at each energy level. Measures that predicted total body skeletal changes did not predict site-specific changes. These data highlight the importance of maintaining lean mass through exercise during periods of ER.

## Introduction

Bone mass is closely associated with total body mass in most healthy individuals with higher body weight associated with greater bone mass, presumably due to greater mechanical loading. Weight loss typically incurs a decline in bone mass; with each 10% reduction in body mass associated with a 1–2% loss of bone mass (Shapses and Riedt, 2006). There remains some controversy about whether it is the lean or fat component of total body mass that is the greater contributor to higher skeletal mass. When controlling for age and height in a cohort of both pre- and post-menopausal women, both fat mass and lean mass were significant independent predictors of total body bone mineral content (BMC; Khosla et al., 1996). In post-menopausal women, some studies indicate that fat mass is a greater predictor of total bone mass compared to lean mass (Compston et al., 1992; Reid et al., 1994; Yang et al., 2015); however, another study demonstrated that lean mass was a better predictor of total bone mass, while fat mass more closely paralleled regional changes in bone mass (Chen et al., 1997). In other populations, lean mass is a better predictor of total bone mass; these include young women (Wang et al., 2005), children (Manzoni et al., 1996), as well as middle-aged males and females (Park et al., 2012). In a meta-analysis of over 40 different studies, lean mass was more often demonstrated to have a greater effect on regional and total body bone mineral density (BMD) than did fat mass (Ho-Pham et al., 2014).

Low energy availability (energy intake – exercise energy expenditure/fat free mass) negatively impacts bone, leading to rapid increases in bone resorption and declines in bone formation directly related to the severity of energy availability (Ihle and Loucks, 2004). Similarly, multiple studies in rodents have demonstrated the detrimental effect of energy restriction (ER) on bone (Hamrick et al., 2008; Devlin et al., 2010; Hawkins et al., 2010; Metzger et al., 2016). Concurrent with the loss in bone mass is a reduction in fat mass and lean mass (Hamrick et al., 2008) as well as suppressed circulating levels of leptin and insulin-like growth factor I (IGF-I) and, in females, estradiol (Ihle and Loucks, 2004; Hamrick et al., 2008; Devlin et al., 2010; Mallinson et al., 2013).

What is less well understood is how exercise during low energy availability impacts body composition and bone parameters. In a tightly controlled study, Ihle and Loucks (2004) had young healthy women expend 15 kcal/kg of lean body mass in exercise energy expenditure daily while under controlled levels of dietary intake. Within 5 days, mild to moderate levels of low energy availability with exercise energy expenditure led to a suppression of IGF-I and a bone formation marker, procollagen type 1 amino-terminal propeptide (P1NP). At a severe level of low energy availability, a decrease in serum estradiol and a concomitant increase in a bone resorption marker, N-terminal telepeptide (NTx), was also observed (Ihle and Loucks, 2004). Due to the challenges of maintaining such a tightly controlled study with human participants, this study was limited to 5 days and, therefore, could not confirm long-term impacts on body composition or bone mass. DiMarco et al. (2007) generated reduced energy availability with exercise by giving female rats access to a running wheel. In this voluntary exercise model, 30% ER resulted in decreased energy availability concurrent with weight loss, decreased serum leptin and estradiol, and decreased femoral and tibial BMC (DiMarco et al., 2007).

Previously, we used a rodent model to examine the impact of ER with and without exercise on metabolic hormones, body composition, and BMD (Metzger et al., 2016). In that study, we measured energy intake and controlled exercise energy expenditure via treadmill running to ensure equivalent energy balance between the exercise and sedentary conditions. Treadmill running during 12 weeks of ER in mature female rats resulted in 12% higher total body BMC and 11% higher volumetric BMD at the proximal tibia metaphysis than in energy-restricted sedentary rats, but had little effect on metabolic hormones.

The purpose of this current study was to determine predictors of both total body BMC and local volumetric BMD of the proximal tibia after short-term (4 week) and long-term (12 week) periods of ER, with and without exercise. Utilizing a rodent model provides the ability for tight control over dietary energy and nutrient intake as well as exercise energy expenditure, allowing for equivalent energy balance across exercise and sedentary groups. Additionally, our animal model allows us to measure changes over much longer periods than is feasible with most human experimental subjects; the current study tracks adaptations for as long as twelve weeks (roughly 12% of a rat’s lifespan). We hypothesized that exercise would preserve lean mass during ER, improve circulating IGF-I levels, and mitigate bone loss. Additionally, we hypothesized that lean mass, fat mass, serum leptin, and serum IGF-I would predict both total and site-specific bone mass across all groups.

## Materials and Methods

### Animals

Five-months-old virgin female Sprague–Dawley rats (*n* = 25–35 per group) were purchased from Harlan Laboratories (Houston, TX, United States) and housed two per cage in a room with 12 h light-dark cycles and allowed free access to food and water. Animals were switched from standard rodent chow (Teklad 2018 non-purified diet) and acclimated to the AIN-93M purified rodent chow (Research Diets D10012M, Research Diets, Inc., New Brunswick, NJ, United States; Reeves et al., 1993a,b). Previous work from our lab (Allen et al., 2001) demonstrated loss of cancellous bone 4 weeks after switching from a Teklad 2018 rat non-purified diet (Harlan-Teklad) to the AIN-93M diet. Therefore, rats were acclimated to the AIN-93M diet for 8 weeks to assure steady-state bone mass prior to the initiation of interventions. Following acclimation, animals were singly housed and block assigned into groups based on proximal tibia vBMD, determined by peripheral quantitative computed tomography (pQCT) scans. The three sedentary groups included (1) *ad libitum* fed sedentary (Adlib + SED), (2) –20% ER sedentary (–20ER + SED), and (3) –40% ER sedentary (–40ER + SED), and the three exercise groups, included (1) *ad libitum* fed with exercise (Adlib + EX), (2) –10% ER with exercise (–10ER + EX), and (3) –30% ER with exercise (–30ER + EX).

For the analyses used in this study, animals from multiple cohorts of a longitudinal study were pooled together to increase statistical power, resulting in 25–35 animals in each of the six groups. The sedentary ER groups (–20ER + SED and –40ER + SED) achieved ER by eating custom-made variations of the AIN-93M diet with energy content of the diet reduced by 20 and 40%, respectively, while 100% of all other essential nutrients were provided, as previously reported (Metzger et al., 2016). The exercising ER groups (–10ER + EX and –30ER + EX) were fed separate versions of the AIN-93M diet with total energy intake reduced by 10 and 30%, respectively, while concurrently increasing their weekly energy expenditure by 10% via treadmill running. Dual energy X-ray absorptiometry (DXA) scans and pQCT scans were completed on anesthetized rats at 4 and 12 week time points and saphenous vein blood draws were performed at the same times to assess serum endocrine factors. All animal procedures were approved by the Texas A&M University Institutional Animal Care and Use Committee conform to the NIH Guide for the Care and Use of Laboratory Animals.

### Dietary Treatment

Both *ad libitum* groups (Adlib + SED and Adlib + EX) consumed the AIN-93M standard purified diet freely. All ER rats voluntarily ate their allotted food. Animals in the –40ER + SED group were given a 40% energy-restricted diet, a modified version of the AIN-93M diet (Research Diets, Inc., New Brunswick, NJ, United States) that contained a higher density of all other essential nutrients to achieve 100% of the rat’s daily dietary requirements (see **Table 1**). The –40ER + SED rats were given 0.61 g of the specially formulated diet for every 1 g eaten by the *ad libitum* fed group, while –30ER + EX rats were given 0.71 g of food for every 1 g eaten by Adlib + SED animals. Rats in the –20ER + SED groups were given 0.81 g of the diet for every 1 g of AIN-93M consumed by the *ad libitum* fed while rats in the –10ER + EX group were given 0.91 g of the specially formulated diet for every 1 g of AIN-93M consumed by the *ad libitum* fed group. Food intake was measured daily (to 0.1 g accuracy) throughout the study to ensure accurate measures of energy intake.

**Table 1:** Formulations of macronutrients of the AIN-93M diet for restriction of energy while maintaining other nutrients at 100%.

Macronutrient (%kcal)	AIN-93M	10% ER	20% ER	30% ER	40% ER
*Protein*	15	16	18	21	24
*Carbohydrate*	76	73	70	66	60
*Fat*	9	10	12	13	16
*Kcal/g*	3.85	3.83	3.81	3.79	3.76


### Energy Expenditure

For this study, daily energy expenditure was assumed to equal daily energy intake, as measured over a minimum of 4 days during each week. This last assumption is based on a number of observations verifying that female rats increase *ad libitum* energy intake voluntarily to match energy output during exercise training protocols (Jennings, 1969; Tokuyama et al., 1982; Anantharaman-Barr and Decombaz, 1989; DiMarco et al., 2007). Exercise trained groups completed treadmill running at a moderate intensity (∼60% VO_2max_) 4 days/week. The running duration was calculated for each rat at the start of each week of the protocol based on the animal’s current body weight and an energy cost for treadmill running of ∼0.22 kcal/kg/min, based on data on virgin female rats from Brooks and White (1978). The goal was to compute that week’s treadmill running time sufficient to produce the desired increase in caloric expenditure (+10% energy intake) as averaged over 1 week. For a rat weighing 295 g (average weight at 6 months of age for virgin female rats), this required ∼60-min training bouts four times per week. The duration of the exercise session was adjusted every week for each animal according to body weight to assure an average 10% weekly increase in the rat’s energy expenditure.

### Treadmill Running

All animals were acclimated to treadmill running on custom-made 12-lane rat treadmill 3 weeks prior to the study while acclimating to the AIN-93M diet. The first week of treadmill acclimation consisted of five 5-min sessions at 15 m/min on a 15% grade. Over the next 2 weeks, rats were exercised three times/week on a 15% grade for incrementally increased duration and speed. By the beginning of the experimental period, all EX groups engaged in moderate intensity treadmill running 4 days/week to increase energy expenditure by 10% averaged over the entire week of training. Running speed was 25 m/min at a 10% grade for 60–100 min. Exercise duration was calculated for each rat weekly based on body weight and documented energy expenditure per g body weight at this speed and grade, which represents ∼60% of maximal oxygen consumption for adult female rats (Brooks and White, 1978).

### Peripheral Quantitative Computed Tomography

After 4 and 12 weeks of the experimental intervention, rats were anesthetized via vaporized isoflurane for *in vivo* measures of the proximal tibia metaphysis (mixed cortical and cancellous bone site) taken on a Stratec XCT Research-M device (Norland Corp., Fort Atkinson, WI, United States). Metaphyseal volumetric bone mineral density (vBMD) was measured at the proximal tibia 4 slices from the growth plate. Three contiguous slices were averaged for one measure at the proximal tibia metaphysis. Scans were completed with 2.5 mm/s scan speed, 100 μm voxel resolution, and 0.5 mm slice thickness. Measures obtained from the *in vivo* pQCT scans include cancellous vBMD and total vBMD (cancellous + cortical shell).

### DXA

Dual energy X-ray absorptiometry scans were performed on rats anesthetized via injected ketamine/dexmedetomidine at week 4 and 12 weeks to determine body composition (lean and fat mass) as well as total skeletal mass (BMC). DXA scans were performed on a GE Lunar Prodigy with small animal software (GE Lunar Prodigy Small Animal Program, GE Healthcare) with the anesthetized animal positioned prone on the table with its long axis aligned to the center of the table.

### Serum Markers

Serum leptin concentrations were measured using ELISA (Alpco Diagnostics, Salem, NH, United States); the inter-assay coefficient of variation was less than 10% and the lowest detectable level was 10 pg/mL. Serum IGF-I concentrations were measured via EIA (Immunodiagnostic Systems Inc., Gaithersburg, MD, United States) with the inter-assay coefficient of variation was 6% and the lowest detectable level was 2.8 ng/mL.

### Statistical Analyses

All data were tested for outliers and homogeneity. Food intake from both Adlib groups was analyzed with an independent samples *t*-test at both 4 and 12 weeks. All other data were analyzed using a 2 × 3 factorial (exercise by energy level) ANOVA to determine main effects of EX and ER. If an exercise-by-energy level interaction was present (*p* < 0.05), all-groups analysis was completed. If the main effects were significant (*p* < 0.05), a Duncan *post hoc* test was used to determine differences among groups. For all measures, *p*-values and effect sizes (partial eta squared) for main effects are reported. Multiple linear regression was completed using total body BMC as the dependent factor and lean mass, fat mass, serum leptin, and serum IGF-I as independent factors. A separate regression was completed with cancellous vBMD as the dependent factor and the same independent factors as previously listed. A new model was accepted when the adjusted *R*^2^ increased and all slopes in the model were significant (*p* < 0.05). If these criteria were not met, the previous model was accepted. Statistical analyses were completed on SPSS (IBM, Armonk, NY, United States). All data are represented as mean ± standard deviation.

## Results

### Body Weight Declined With Energy Restriction, But Was Increased With Exercise in Energy-Restricted Groups

**FIGURE 1:**
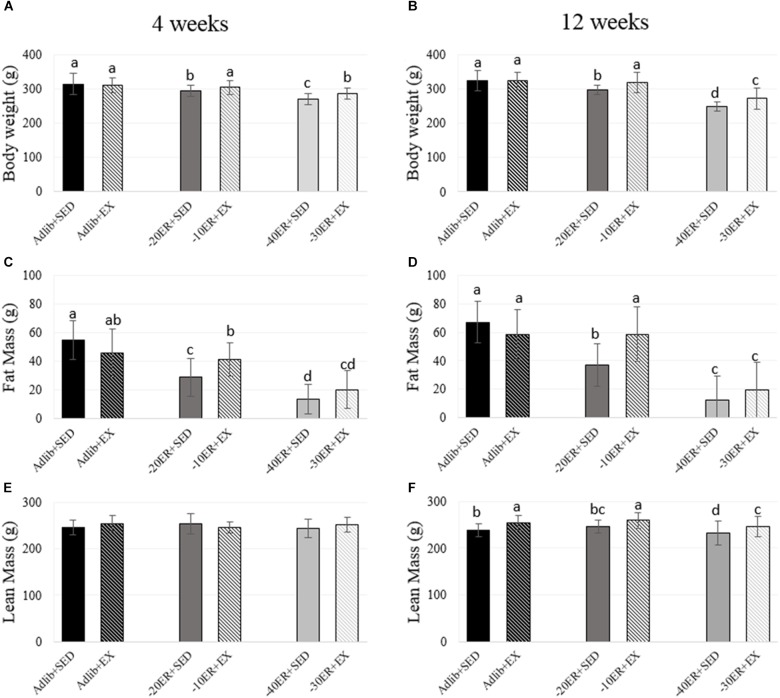
Bodyweight, fat mass, and lean mass at 4 and 12 weeks. Bars not sharing the same letter are statistically different, *p* < 0.05. **(A)** Energy restriction resulted in lower body weight in all energy-restricted conditions. Exercise led to higher bodyweight than energy-matched sedentary animals. **(B)** –20ER + SED and –40ER + SED had progressively lower bodyweight compared to Adlib + SED. EX in both ER conditions increased bodyweight. **(C)** Fat mass was lower with each grade of ER. Adlib + EX decreased fat mass compared to Adlib + SED while exercise during ER increased fat mass. **(D)** Fat mass was lower with ER. In Adlib + EX vs. Adlib + SED, exercise decreased fat mass while exercise in both ER conditions increased fat mass. **(E)** There were no statistical differences in lean mass. **(F)** Lean mass increased with exercise in all conditions.

There were no baseline differences in food intake at the beginning of the study (all rats consumed an average of 17.49 g ± 2.4). All rats within the study completed assigned running in EX groups, achieving an estimated 10% increase in weekly energy expenditure. All ER rats consumed all food supplied. At both 4 and 12 weeks, Adlib + EX rats had higher average daily food intake compared to Adlib + SED (*p* = 0.02 for both time points; **Table 2**). Therefore, we confirmed that Adlib + EX rats increased energy intake due to exercise (8.7% greater at week 4 and 9.5% greater at week 12) while body weights between these two groups were not different at either time point. Comparing all groups, there was an exercise-by-energy interaction (*p* = 0.016, effect size = 0.036) at 4 weeks. There was also a main effect of energy status on body weight (*p* < 0.0001, effect size = 0.349) and a main effect of exercise (*p* = 0.003, effect size = 0.038). At 4 weeks, body weight in –40ER + SED was lower than –30ER + EX and –20ER + SED was lower than –10ER + EX, with Adlib + SED, and Adlib + EX having the highest values (**Figure 1**). At 12 weeks, there was an energy-by-exercise interaction (*p* = 0.018, effect size = 0.046) as well as main effects of both energy status (*p* < 0.0001, effect size = 0.564) and exercise (*p* < 0.0001, effect size = 0.073; **Figure 1**). In general, exercised energy-restricted rats maintained body weight better than their sedentary counterparts.

**Table 2:** Adlib + SED and Adlib + EX average daily food intake at 4 and 12 weeks.

Group	4 weeks	12 weeks
*Adlib + SED*	14.9 ± 1.4	13.6 ± 1.3
*Adlib + EX*	16.2 ± 1.3^∗^	14.9 ± 1.9^∗^


*^∗^Indicates difference from Adlib + SED in *t*-test (*p* < 0.05).*

### Both 4 and 12 Weeks of Energy Restriction Caused Significant Reductions in Fat Mass, While Total Skeletal Mass (BMC) Declined Only After 12 Weeks

**FIGURE 2:**
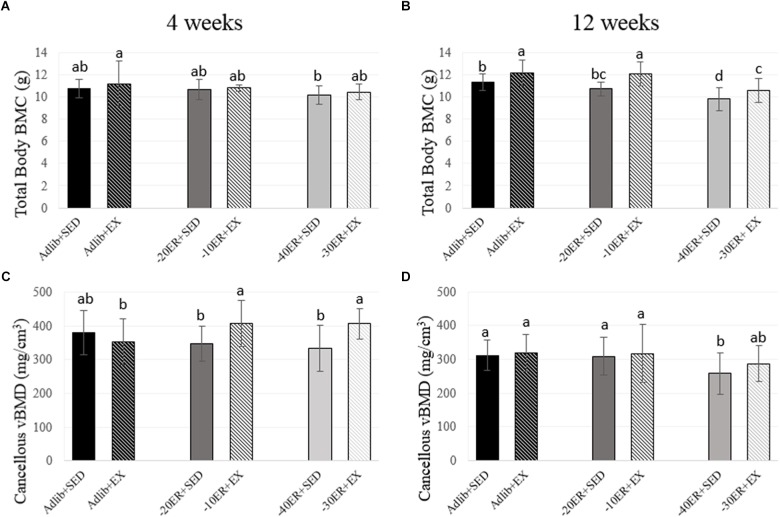
Total body bone mineral content and proximal tibia metaphysis volumetric bone mineral density at 4 and 12 weeks. Bars not sharing the same letter are statistically different, *p* < 0.05. **(A)** There is a trend for an effect of energy on bone mineral content. **(B)** In the –40ER + SED and –30ER + EX conditions, BMC was lower than Adlib + SED. In all conditions, exercise resulted in higher BMC compared to energy-matched sedentary. **(C)** Exercise improved vBMD compared to energy-matched controls in both ER groups. **(D)** –40ER + SED had lower vBMD compared to all other groups.

Exercise during ER resulted in greater lean mass and BMC at 12 weeks. At week 4, there was a main effect of energy status on fat mass (*p* < 0.0001, effect size = 0.534). Fat mass was lower in all ER groups compared to Adlib groups and EX during ER resulted in slight increases in fat mass (**Figure 1**). There were no main effects for lean mass, but there was a trend for a main effect of energy status on total body BMC (*p* = 0.05, effect size = 0.073; **Figure 2**). At 12 weeks, there was an interaction between energy status and exercise (*p* = 0.002, effect size = 0.123) and a main effect of energy status on fat mass (*p* < 0.0001, effect size = 0.597). Both –40ER + SED and –30ER + EX groups had a lower fat mass than all other groups and –20ER + SED was lower than –10ER + EX and both *ad libitum* fed groups. There were main effects of both energy status and exercise on lean mass (*p* = 0.021, effect size = 0.076; *p* < 0.0001, effect size = 0.154) and total body BMC (*p* < 0.0001, effect size = 0.338; *p* < 0.0001, effect size = 0.244). At 12 weeks, exercise lead to a significant increase in lean mass of ∼ 6% compared to sedentary energy-matched controls at all energy levels (**Figure 1**). Exercised animals also exhibited a higher total body BMC (∼+7–12.5%) compared to sedentary controls at all levels of energy status (**Figure 2**).

### Energy Restriction Led to a Decline in Cancellous vBMD at the Proximal Tibia; However, Exercise During ER Prevented Losses at 4 Weeks and Mitigated Losses at 12 Weeks

At week 4, there was an energy-by-exercise interaction (*p* = 0.007, effect size = 0.119) and a main effect of exercise on cancellous vBMD at the proximal tibia metaphysis (*p* = 0.01, effect size = 0.08). The –30ER + EX group and –10ER + EX group were not different from Adlib + SED, but higher than all other groups (**Figure 2**). The main effect of exercise remained at 12 weeks (*p* = 0.006; effect size = 0.074) with –40ER + SED the lowest of all groups and –30ER + EX not different from any of the other groups. At 12 weeks, there was a main effect of energy on cancellous vBMD (*p* = 0.041, effect size = 0.067) and a non-statistical effect of exercise (*p* = 0.065; **Figure 2**). There were no effects on total vBMD (cortical + cancellous) at the proximal tibia metaphysis at either 4 or 12 weeks (data not shown).

### Serum IGF-I Concentrations Declined Most Significantly in the Highest Energy Restriction Groups Regardless of Exercise Status

**FIGURE 3:**
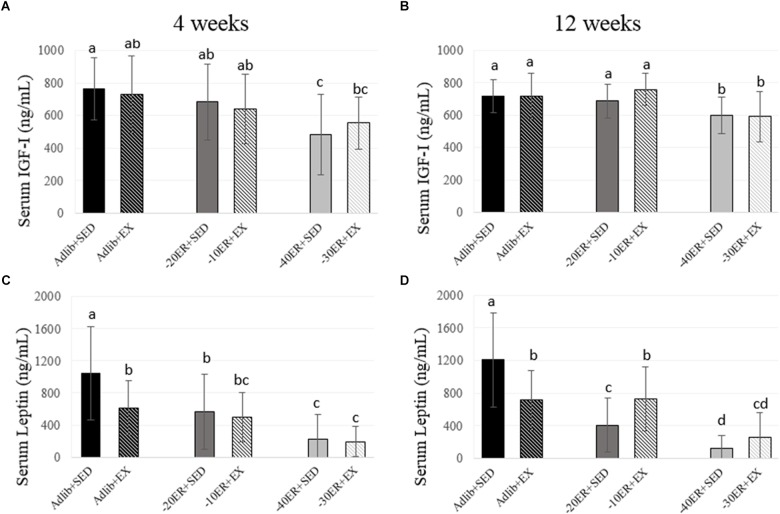
Serum IGF-I and leptin concentrations at 4 and 12 weeks. Bars not sharing the same letter are statistically different, *p* < 0.05. **(A)** At 4 weeks, serum IGF-I concentrations were lower in –40ER + SED and –30ER + EX. **(B)** At 4 weeks, serum leptin concentrations due to exercise in *ad libitum* and due to ER. **(C)** At 12 weeks, serum IGF-I concentrations were lower only in the –40ER + SED and –30ER + EX groups. **(D)** At 12 weeks, serum leptin concentrations were lower due to ER. In Adlib + EX, leptin was lower than Adlib + SED, but in the ER groups, exercise led to higher leptin concentrations.

There was a main effect of energy status at 4 weeks (*p* = 0.001, effect size = 0.169). Adlib + SED had the highest serum IGF-I concentration and –40ER + SED had the lowest concentration. At 12 weeks, there was a significant main effect of energy status (*p* < 0.0001, effect size = 0.196) and a non-statistical difference of exercise (*p* = 0.061). Both –40ER + SED and –30ER + EX groups had lower concentrations than all other groups (**Figure 3**).

### Serum Leptin Concentrations Declined Due to Exercise in *ad libitum* Fed Rats and Due to ER in All Rats, Paralleling Losses in Fat Mass

There was a main effect of both energy status and exercise on serum leptin concentrations after 4 weeks (*p* < 0.0001, effect size = 0.309; *p* = 0.04, effect size = 0.052). Adlib + SED had the highest leptin concentrations followed by Adlib + EX with declines in circulating leptin in all ER groups (**Figure 3**). At 12 weeks, there was an energy-by-exercise interaction (*p* < 0.0001, effect size = 0.186) and a main effect of energy status (*p* < 0.0001; effect size = 0.419). Adlib + SED had the highest concentrations of serum leptin followed by Adlib + SED and –10ER + EX. –20ER + SED was lower than –10ER + EX but not different than –30ER + EX. Leptin concentrations were lowest in –40ER + SED (**Figure 3**).

### At Both 4 and 12 Weeks, Fat Mass and Lean Mass Were Significant Predictors of Total Body Bone Mineral Content

**FIGURE 4:**
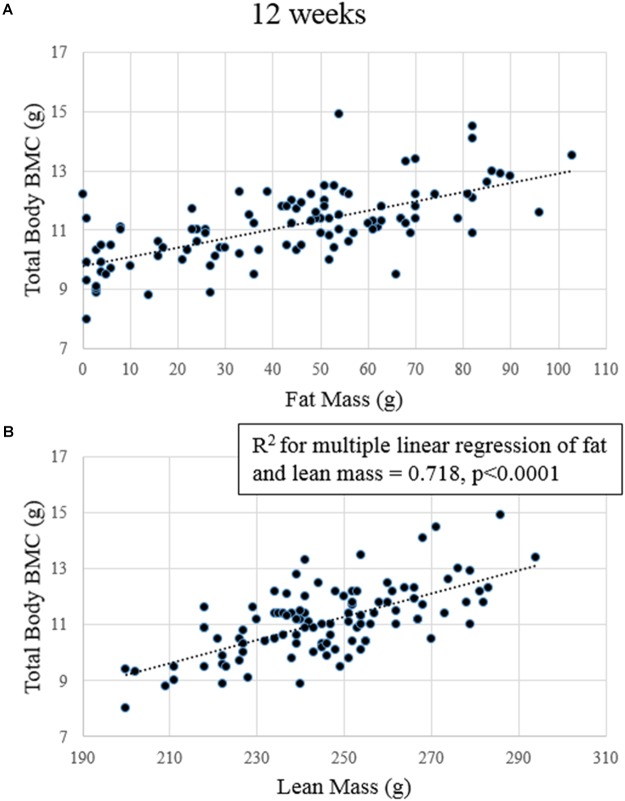
Regression plots of lean mass and fat mass to total body BMC after 12 weeks. **(A)** Fat mass significantly predicted BMC. **(B)** Lean mass significantly predicted BMC.

After 4 weeks, fat mass, lean mass, and serum IGF-I concentrations predicted total body BMC (*p*-values for model slopes = 0.001, < 0.0001, and 0.042, *R*^2^ = 0.538, *R*^2^ adjusted = 0.515; data not shown). No variables significantly predicted cancellous vBMD or total vBMD. At 12 weeks, fat and lean mass were significant predictors of total body BMC (*p*-values for model slopes ≤ 0.0001 for both *R*^2^ = 0.718 and *R*^2^ adjusted = 0.707; **Figure 4**).No variables predicted cancellous or total vBMD at the proximal tibia at 12 weeks.

## Discussion

This study provides new information regarding the impact of exercise during ER. Primary findings of this study include (1) exercise during ER increases lean mass as well as total body skeletal mass, (2) both fat and lean mass are strong predictors of total body BMC during short-term and long-term ER, and (3) proximal tibia cancellous BMD was not predicted by any of the variables analyzed (fat mass, lean mass, serum IGF-I concentrations, and serum leptin concentrations). Therefore, factors like lean mass and fat mass predicted total skeletal mass at both 4 and 12 weeks, but not that of a local bone site. However, exercise still exerted a positive effect on metaphyseal cancellous volumetric BMD, likely due to increased mechanical loading.

One of the strengths of this study is the large cohort size (*n* = 25–35 per group), allowing for strong statistical comparisons and regression analyses. Additionally, using an animal model with precise matching of energy balance in both sedentary and exercise groups allows a fair test of the value of exercise in the context of ER, with respect to effects on body composition, bone outcomes, and circulating metabolic factors. In our study, measures of lean mass, total body BMC, and proximal tibia cancellous vBMD were all higher in exercising animals vs. sedentary rats at matched levels of energy balance. This is one of the first studies to clearly examine the impact of exercise on body composition and skeletal mass at various levels of ER in matched sedentary and exercising groups.

More specifically, exercise during long-term ER (12 weeks) increased lean mass in our rats compared to energy-matched sedentary rats despite losses in fat mass. Losses in fat and lean mass due to ER have been documented in similar animal models (Hamrick et al., 2008). In humans, weight loss regimens solely utilizing ER often lead to loss of lean mass as well as loss of fat mass (Weiss et al., 2007). In our study, rats with the highest ER (–40%) experienced an 81% decline in fat mass compared to sedentary *ad libitum* controls and a 2.6% reduction in lean mass. Human clinical trials have also demonstrated a preservation of lean mass when exercise is part of the weight loss program (Weiss et al., 2007). In our study, we found that exercise improved lean mass by 5.4–6.5% at all levels of energy balance.

After 4 weeks ER, the sedentary –40% rats in this study had 5% lower total body BMC compared to *ad libitum* fed sedentary rats. By 12 weeks of –40% ER, the sedentary ER group had 14% lower total body BMC and 12% less cancellous vBMD at the proximal tibia. These changes were concurrent with reductions in body weight, fat mass, and lean mass. Similarly, in a randomized controlled trial, 2 years of 25% ER in non-obese adults resulted in significantly lower BMD in the lumbar spine and hip regions compared to the *ad libitum* group as well as lower body weight, fat mass, and fat free mass (Villareal et al., 2016). This underscores the detrimental effect of ER without concurrent exercise on both total body BMC and site-specific vBMD, especially during long-term ER.

In human trials, weight loss due solely to caloric restriction causes losses in BMD in clinically relevant fracture sites; however, exercise-induced weight loss did not result in decrements in BMD at the same sites (Villareal et al., 2006). In our study, we found that exercise during ER resulted in 7–12% greater total body BMC across all levels of energy status after 12 weeks. At the proximal tibia metaphysis, exercise resulted in 2.5–3% higher cancellous vBMD compared to energy-matched controls in both the *ad libitum* fed and moderate ER (–20ER + SED and –10ER + EX) groups after 12 weeks of intervention. Interestingly, exercise seemed to have the greatest effect in the –30% ER + EX group, with 11% higher cancellous vBMD compared to energy-matched controls (–40ER + SED). At 12 weeks, exercise in the –10% ER group prevented bone loss and resulted in 6% higher total body BMC compared to *ad libitum* fed sedentary rats; however, at –30% ER, while exercise mitigated but did not prevent the loss in bone (7% higher than –40 + SED but 7% lower than *ad libitum* fed sedentary). Therefore, exercise coupled with mild/moderate ER may be able to prevent bone loss, but may only mitigate the loss during more severe ER as commonly utilized during weight-loss regimens by overweight humans.

In this study, we also examined circulating leptin and IGF-I concentrations as potential metabolic markers of ER that may predict bone outcomes. Leptin concentrations decline in parallel with loss of fat mass, while IGF-I concentrations are suppressed by ER (Ihle and Loucks, 2004; Hamrick et al., 2008; Devlin et al., 2010). IGF-I is anabolic to bone and circulating IGF-I concentrations directly impact bone growth and density (Yakar et al., 2002; Giustina et al., 2008). Leptin has equivocal roles in regulating bone mass, but, in the case of ER, low circulating concentrations may suppress bone anabolic processes (Hamrick, 2004). In the present study, circulating IGF-I and leptin concentrations were significantly lowered by ER, particularly in the most severe ER groups. With both of these metabolic hormones, energy status had more of an effect on circulating concentrations compared to exercise.

There are conflicting data on the impact of exercise on circulating IGF-I concentrations. In trained runners, several days of ER to 50% of estimated energy requirements and intensive treadmill running resulted in a 17% decline in circulating IGF-I concentrations, but there was no change in serum IGF-I concentrations due solely to the exercise regimen in runners in energy balance (Zanker and Swaine, 2000). In a cohort of young females, serum IGF-I concentrations were higher (up to 107% higher) in those who were physically active compared to sedentary women (Ardawi et al., 2012). We previously demonstrated that, in rats subjected to 12 weeks of 40% ER, exercise did not mitigate the reductions in IGF-I concentrations observed in energy-matched sedentary controls (Metzger et al., 2016). In the present study, there was a trend for an exercise effect at 12 weeks in serum IGF-I concentrations, but that positive effect seems to be limited to the moderate (–10%) ER exercised animals. Taken together, these studies demonstrate that the main contributor to changes in circulating IGF-I concentrations is energy status rather than exercise.

One of the primary goals of this study was to address what factors could predict total body and site-specific bone outcomes. Using multiple linear regression, we tested lean mass, fat mass, and serum IGF-I and leptin concentrations on either total body BMC or proximal tibia vBMD. At 4 weeks, we found that fat mass, lean mass, and serum IGF-I concentrations together predicted approximately 54% of the variability in total body BMC. At 12 weeks, only fat and lean mass predicted total body BMC together explaining approximately 72% of the total variability. Surprisingly, even though leptin was positively correlated with fat mass (*r* = 0.618 at 4 weeks and *r* = 0.691 at 12 weeks), it was not predictive of bone outcomes at any time point, even though fat mass was a strong independent predictor. Contrary to our hypothesis, no factor we measured predicted site-specific vBMD at the proximal tibia. The results of this study in a rodent model are consistent with many studies in humans showing the strong association between fat mass, lean mass, and total bone mass (Ho-Pham et al., 2014). Contrary to studies that have found lean mass or fat mass to be a stronger predictor than the other, in our study, both were equally strong and together predicted a significant portion (up to 70%) of total body BMC. Some studies have found correlations between fat or lean mass and specific parts of the skeleton like the hip (Hootkouper et al., 1995; Reid et al., 1995; Mallinson et al., 2013); however, changes in vBMD at our local bone site, the proximal tibia, were not closely associated with any of the variables we tested.

While fat mass predicts bone outcomes in many human clinical studies as well as in our rats, there appears to be a point at which high body fat mass is actually detrimental to bone where higher fat mass correlates with low bone mass and increased fracture risk (Hsu et al., 2006). Therefore, the positive correlations between fat mass and bone mass may only exist within underweight and normal-weight populations. Lean mass as a predictor of total body bone outcomes during ER may have translational value in health conditions that cause weight loss. One such example is breast cancer survivors where both fat and lean mass were correlated with total body BMD (George et al., 2013). For this population and others with related conditions, exercise therapy with the goal of increasing lean mass may be a valuable treatment to mitigate bone loss and fracture risk.

Prolonged, severe ER like that seen in individuals diagnosed with the Female Athlete Triad (Nattiv et al., 2007) may result in significant bone loss even with high levels of exercise training. For example, one study using high-resolution peripheral quantitative computed tomography (HR-pQCT) to assess vBMD in the weight bearing tibia determined that amenorrheic athletes had lower bone mass than non-athletic controls, despite their regular weight-bearing activity (Ackerman et al., 2011). One important mediator is disrupted reproductive hormone levels and cyclicity in these athletes (Mallinson et al., 2013). Therefore, weight-bearing exercise cannot fully compensate for the deleterious effects of severe or long-term ER.

Limitations of this study include lack of ability to measure full endocrine status of these rats, including female sex hormones. Additionally, due to the large *n* comparisons of this study, we did not have matching histological measures of bone turnover (bone formation rate and osteoclast surfaces) for each animal nor measures of mechanical properties. Finally, some limitations within the actual study design should be addressed. There is a potential increase in stress caused by treadmill running although this was lessened as much as possible by not utilizing the shock grid. Additionally, measures of in-cage activity were not completed in this study; therefore, we do not know if EX or ER lead to an increase in overall cage activity which may have additionally impacted study outcomes.

## Conclusion

This study demonstrated that, after both short- and long-term ER achieved with and without exercise, fat and lean mass are the strongest predictors of total body BMC. Exercise resulted in higher lean mass throughout all levels of energy balance and this increased lean mass was positively associated with higher BMC. With moderate ER (–20% in sedentary, –10% in exercising), concurrent exercise fully prevented bone loss and, with severe ER (–40% in sedentary, –30% in exercising), it significantly mitigated loss of skeletal mass. This positive impact of exercise during prolonged ER appears to be due to in part to the better maintenance of lean mass. Therefore, exercising to maintain lean body mass during periods of ER might prove to be an important therapeutic tool to protect bone mass.

## Author Contributions

CM compiled, analyzed, and interpreted the data and drafted and edited the manuscript. SS and KB ran the animal protocol, performed data analyses, and edited the manuscript. MDS and SB designed the original protocol and edited the manuscript. CM, SS, KB, MDS, and SB approved the final version.

## Conflict of Interest Statement

The authors declare that the research was conducted in the absence of any commercial or financial relationships that could be construed as a potential conflict of interest.
